# Predictors of successful transition of adolescents and young adults living with HIV from pediatric to adult-oriented care in southern Ethiopia: a retrospective cohort study

**DOI:** 10.1186/s12913-024-11319-y

**Published:** 2024-07-24

**Authors:** Mulugeta Shegaze Shimbre, Gelila Abay, Abebe Gedefaw Belete, Melkamu Merid Mengesha, Wei Ma

**Affiliations:** 1 School of Public Health, Cheeloo College of Medicine, Shandong University, Jinan, 250012 Shandong China; 2 School of Public Health, College of Medicine and Health Sciences, Arba Minch Universit, Arba Minch, Ethiopia; 3 Department of Health Sciences, Faculty of Medicine, Lund University, Lund, Sweden

**Keywords:** Successful transition, Predictors, Adolescents and young adults living with HIV, Southern Ethiopia

## Abstract

**Background:**

The introduction of highly active antiretroviral therapy has significantly improved the life expectancies of children and adolescents living with HIV, leading to an increased number transitioning to adult care. However, there has been a lack of studies in Ethiopia focusing on factors influencing the success of this transition. Therefore, this study aimed to determine predictors of a successful transition from pediatric to adult HIV clinics among adolescents and young adults living with HIV in health facilities in southern Ethiopia.

**Methods:**

A retrospective cohort study included 337 adolescents and young adults who transitioned to adult-oriented HIV care. Successful transition was defined as having a viral load of less than 1000 copies/ml and maintaining care during the first year post-transition. Patients’ antiretroviral therapy (ART) cards and monitoring charts were reviewed. Secondary data analysis was conducted using a multivariable binary logistic regression model to identify predictors of a successful transition. Using the variance inflation factor, we checked for multi-collinearity between variables and assessed model fitness with the Hosmer and Lemeshow goodness-of-fit test. Adjusted Odds Ratio (AOR) with 95% confidence intervals (CI) and P-value ≤ 0.05 measured the strength of association and statistical significance.

**Results:**

Of 337 participants, 230 (68.25%) successfully transitioned (95% CI = 63.25, 73.25). Transitioning at age 18 or older (AOR = 4.25; 95% CI = 2.29, 7.87), residing in an urban area (AOR = 1.78; 95% CI = 1.04, 3.02), and being on antiretroviral therapy for more than two years (AOR = 4.25; 95% CI = 1.17, 4.94; *P* < 0.017) were identified as positive predictors and opportunistic infection (AOR = 0.34; 95% CI = 0.15, 0.75; *P* < 0.008) was identified as a negative predictor for a successful transition from pediatric to adult ART clinic.

**Conclusion:**

This study sheds light on the challenges faced by HIV patients transitioning from pediatric to adult care, with less than 70% successfully navigating this critical phase. Factors such as age at transition, residence, duration of ART, and the presence of opportunistic infections were identified as key predictors of successful transition. The findings underscore the urgent need for tailored interventions, including standardized transition plans that address age and urban/rural disparities, to enhance transition outcomes for adolescents and young adults living with HIV in the region.

## Introduction

The proportion of adolescents and young adults living with HIV is increasing globally, with around 85% of these cases occurring in sub-Saharan Africa (SSA). This region is disproportionately affected by the HIV epidemic due to various factors, including limited access to healthcare, education, and prevention services [1].

Following the introduction of highly active antiretroviral therapy (HAART), the life expectancies of HIV-infected children and adolescents have significantly improved. Consequently, a high proportion of adolescents and young adults have transitioned from pediatric to adult HIV care [2]. Transition is described as “a purposeful, planned process that addresses the medical, psychosocial, and educational needs of adolescents and young adults with chronic physical and medical conditions as they move from child-centered to adult-oriented health care systems.“ [3].

A successful transition for adolescents and young adults living with HIV from adolescent to adult-oriented clinics is crucial for their long-term health outcomes. The transition involved a complex process that requires a gradual, individualized approach [4]. This transition involves not only a change in healthcare providers but also a shift in the approach to care, as adult-oriented clinics may have different protocols and expectations. The success of the transition process significantly impacts the outcomes of antiretroviral treatment (ART) and HIV care for individual patients and the overall HIV program in a nation [5, 6]. Successful transitions occur when patients understand and accept their illness, orient themselves toward future goals, and are able to improve their health, independently manage their disease, and assume adult roles and functioning [7, 8]. However, the transition is influenced by a range of factors, including emotional and psychological burden, HIV stigma, and the need for social support and skills development [9].

Comprehensive support during this transition period helps ensure that adolescents continue to receive the necessary medical care, adhere to their treatment regimens, and address any psychosocial challenges they may face. Additionally, communication between healthcare providers, adolescents, and their families empowers them to actively participate in their healthcare decisions, enhancing the overall quality of care. However, the lack of provider preparation, adolescent readiness, comprehensive support, and communication between healthcare providers, adolescents, and their families before transition can impede the success of the transition. Unsuccessful transition may lead to poor retention, loss of follow-up from care, a decline in immune status, unsuppressed viral load, increased morbidity and mortality, and an increased likelihood of secondary HIV transmission [6, 8].

The Joint United Nations Programme on HIV/AIDS (UNAIDS) introduced the 95-95-95 targets, aiming to diagnose 95% of all HIV-positive individuals, provide ART for 95% of those diagnosed, and achieve viral suppression for 95% of those treated by 2030 [10]. Ensuring the effective transition of adolescents living with HIV from pediatric to adult care is a primary concern in achieving these goals [11].

Health facilities in Ethiopia encounter difficulties in addressing the unique requirements of adolescents and young adults living with HIV, such as issues related to adherence to treatment and appointment schedules [12]. A situational analysis of 218 facilities across 23 sub-Saharan African countries, including Ethiopia, revealed that merely 10% of these facilities offered adolescent-friendly HIV treatment and care services [13]. Adolescents and young adults living with HIV in the region reported discomfort and a lack of privacy due to the common practice of providing adolescent care services within the same clinic space as adult patients. This lack of specialized services tailored to the unique needs of adolescents and young adults living with HIV contributes to suboptimal health outcomes in this population.

In Africa, particularly in Ethiopia, where a significant portion of individuals living with HIV are adolescents and young adults, challenges in the transition process have led to unsuccessful transitions [5, 14–16]. These challenges stem from limited access to healthcare, lack of accurate information, insufficient support, and inadequate involvement of adolescents and young adults in their care. Societal and cultural barriers, including stigma and privacy concerns, further exacerbate these issues. Despite the support from healthcare providers regarding adherence, retention in care, and psychosocial support before transition, transitioning adolescents and young adults living with HIV in Ethiopia remains a complex process due to the lack of clear implementation guidelines [14]. In addition, health system-related factors such as unspecified age of transition, disclosure status, and insufficient staff training hinder successful transitions [14, 17]. There is a lack of comprehensive studies addressing the factors influencing successful transitions, prompting further investigation [14]. Moreover, the absence of consensus on the definition and components of a successful transition program impedes consistent reporting of transition principles [18]. Therefore, this study aimed to identify predictors of successful transition into adult HIV clinics among adolescents and young adults living with HIV in southern Ethiopia.

## Methods and materials

### Study design and settings

A retrospective cohort study was conducted in Southern Ethiopia, encompassing the Wolaita, Gamo, Gofa, Knonso, and South Omo Zones, from January 2017 to August 2022. The study was carried out in public health facilities that provide ART services in a region known for its diverse linguistic and ethnic populations. The region has a population of 7,715,891 and covers an area of 93,800 km² [19, 20]. In 2023, according to the Ethiopian Ministry of Health, there are 4,200 adolescents and young adults living with HIV on ART in the region [21]. ART services are available in both pediatric and adult HIV clinics. Adolescents and young adults receive treatment in pediatric clinics until they transition to adult clinics, where they continue their therapy [21, 22]. Despite the availability of general pediatric health care services in every health facility in Southern Ethiopia, there is limited provision of ART services. The majority (88.9%) of the population lives in rural areas, facing challenges such as limited access to education, and high dropout rates. The region also experiences high fertility rates, neonatal mortality, infant mortality, and under-five mortality, alongside high poverty prevalence, low literacy rates, limited media exposure, inadequate sanitation access, and poor hygiene practices [23, 24].

### Population

The study included all eligible adolescents and young adults aged 10–26 in 14 selected health facilities in southern Ethiopia that provide ART services. These individuals transitioned from pediatric to adult-oriented HIV care between January 2017 and August 2022. The records were reviewed from September to October 2023. The study population consisted of those who made this transition during the specified period. The missing key variables from the patient’s records could result in misinterpretation or bias. Therefore, incomplete data were excluded to maintain the accuracy and reliability of the study results. The total sample size was 339. After excluding 2 cases due to incomplete viral load data and transition period information, the final study sample comprised 337 adolescents and young adults who transitioned to adult-oriented HIV care.

### Study variables

The study assessed the success of the transition as the dependent variable. The independent variables included socio-demographic and behavioral factors (age, sex, residence history of smoking and alcohol intake), immunological and clinical conditions (line of ART regimen, WHO staging, follow-up model, and duration of time on ART before transition), and clinical complications and comorbidities (drug side effects, opportunistic infections, and developmental illness).

**A successful transition** is defined as having a viral load of less than 1000 copies/ml within 6–12 months after transitioning and maintaining care by attending each scheduled visit within 12 months post-transition. Conversely, an unsuccessful transition is indicated by a viral load exceeding 1000 copies/ml during the first year after transition or not being retained in care (loss to follow-up or death) within the initial post-transition year [25].

**Retention on HIV care** remaining on treatment without missing a scheduled care visit for 12 months after the initiation of antiretroviral therapy in adult-oriented HIV care [26]. remaining on treatment without missing a scheduled care visit for 12 months after the initiation of antiretroviral therapy in adult-oriented HIV care.

**Adolescents** are a group of people who are between 10 and 19 years old [14].

**Age at transition** the age at which individuals move from pediatric to adult-oriented HIV care, with the study distinguishing between those transitioning at less than 18 years old and those transitioning at 18 years or older.

**Duration on ART** the length of time an individual has been on Antiretroviral Therapy before transitioning to adult-oriented HIV care.

Developmental Disorders refer to health conditions or disorders that affect the physical, cognitive, emotional, or social development of an individual. Examples include autism spectrum disorder, attention deficit hyperactivity disorder (ADHD), intellectual disabilities, and language disorders [27, 28].

First-line ART: In Ethiopia, the recommended first-line regimen for adolescents and young adults (> 10 years of age or > 30 kg body weight) consists of a once-daily dose of Tenofovir (TDF) + Lamivudine (3TC) + Dolutegravir (DTG). These regimens are chosen for their simplified administration, reduced toxicity, and superior efficacy in achieving rapid viral suppression [29].

Second-line ART: In cases where NNRTI-containing regimens were utilized as first-line ART, a combination of a boosted Protease Inhibitor (PI) + two Nucleoside Reverse Transcriptase Inhibitors (NRTIs) is recommended as the preferred strategy for second-line ART among adolescents and young adults. However, if DTG was not utilized in the first-line regimen, a combination of two NRTIs + DTG can be considered as an alternative second-line regimen.

The follow-up model is follow-up appointments are typically scheduled every one, three, or six months, depending on the stability of the patient’s condition and their treatment adherence.

**Lost to follow-up** considered when an individual has failed to visit ART clinics for more than 90 days after the last appointment date and has not been classified as either “died” or “transferring out [26].

**Opportunistic infections (OIs)**Opportunistic infections are infections that are rare in individuals with a healthy immune system but occur more frequently in those with compromised immune function. These infections are typically caused by organisms of low pathogenicity and can lead to severe disease in immunocompromised individuals. Common opportunistic infections in HIV patients include pneumonia, candidiasis, meningitis, and tuberculosis [30, 31].

**Residence** the location where individuals live, categorized as either urban or rural in the context of this study.

**Young adults** are a group of people who are between 18 and 26 years old [32].

### Data collection tools and procedures

The data collection tool was developed by reviewing patients’ ART cards, and monitoring charts and by reviewing related works of literature [2, 3, 5, 14, 15]. The tool includes socio-demographic and behavioral factors like: (age, sex, residence, history of smoking and alcohol intake), immunological and clinical conditions like: (line of ART regimen, WHO Staging, follow-up model, duration of time on ART before transition) and clinical complications and comorbidities like: (Drug side effects, OIs, and Developmental illness). Individual patient ART follow-up cards including monitoring charts were the source of the data. The data were collected by trained data collectors with independent monitors who supervised the data collectors who had a bachelor’s degree in health science had been trained in HIV care management and were working in HIV clinics. The data collection tool was pretested in three HIV care follow-up centers near the study area and corrected accordingly. Three days of training on the objective of the study, content, ethical procedures, and methods of data collection were provided to data collectors. The supervision and periodic review were done at each step of data collection by the supervisor and principal investigator to check for completeness of the information before data analysis.

### Data analysis

The data were collected using Kobo Collect and then exported to Stata SE 18 for data cleaning and statistical analysis. Descriptive analysis, including median with standard deviation, frequency, and percentages, was employed to describe various variables. Bivariable and multivariable binary logistic regression models were utilized to identify predictors of a successful transition.

In the bivariable analysis, independent variables with a P-value ≤ 0.25 were included in the final multivariable model for further analysis [33, 34]. To ensure the reliability of the results, multi-collinearity between variables was assessed using the variance inflation factor (VIF), with a median VIF cut-off point of < 5.

Model fitness was examined using the Hosmer and Lemeshow goodness of fit, with a p-value ≥ 0.05 considered acceptable. The strength of association and statistical significance were evaluated using Adjusted Odds Ratio (AOR) with 95% confidence intervals (CI) and a P-value ≤ 0.05.

## Results

### Socio-demographic, behavioral, clinical, and immunological characteristics

The analysis included data from 337 adolescents and young adults who transitioned to adult-oriented HIV care, due to incomplete viral load data and transition period information. The median age of the study participant during the study enrollment was 15, with an interquartile range (IQR) of ± 4, while the median age at transition was 19 years, with an IQR of ± 4. The study participants had a minimum of age 14 and a maximum of 25 years. Regarding time since diagnosis (in years), the median time since diagnosis was 9 years, with IQR of ± 6. The majority (68.25%) of the individuals were urban dwellers. Additionally, around 5.64% of the clients had a history of alcohol intake. Among the total patients, less than 1% experienced drug side effects. About 10% of them had opportunistic infections, and a minor percentage, specifically 1.78%, reported developmental disorders. Among the study participants, approximately 56.06% had been on antiretroviral therapy (ART) for more than two years. Furthermore, a substantial majority, comprising around 91%, were following a first-line ART regimen (**refer to** Table 1).

**Table 1:** Baseline sociodemographic, behavioral, clinical, and immunological characteristics of adolescents and young adults living with HIV who transition from pediatric to adult-oriented HIV care in selected health facilities of southern Ethiopia (*n* = 337)

Variable	Categories	*n*	%	Pearson’s chi2	*P*-value
Age at Transition	Median(IQR) 19 ± 4				
Age during the study enrollment	Median(IQR) 15 ± 5				
Time since diagnosis (in years)	Median(IQR) 9 ± 6				
Residence	Urban Rural	230 107	68.25 31.75	10.7235	0.001
Gender	Male Female	162 175	48.07 51.93	2.3634	0.124
Alcohol Intake during the study enrollment	Yes No	19 318	5.64 96.36	0.0003	0.987
Smoking during the study enrollment	Yes No	18 319	5.34 94.66	0.1385	0.710
Drug side effects	Yes No	2 335	0.59 99.41	0.9360	0.333
Opportunistic infections during study enrollment	Yes No	34 303	10.09 89.91	7.8360	0.005
Developmental disorder	Yes No	6 331	1.78 98.22	0.0071	0.933
Follow-up model	Every 1 month Every 3 month Every 6 month	124 89 124	36.8 26.4 36.8	3.1494	0.207
Duration on ART(before transition)	< 1 year Between 1 & 2 year ≥ 2 year	62 102 173	18.40 30.27 51.34	6.9420	0.031
ART regimen	First line Second line	304 33	90.21 9.79	0.0354	0.851
WHO staging	Stage I Stage II Stage III & IV	225 55 57	66.77 16.32 16.91	1.2693	0.530

### Predictors of successful transition from pediatrics to adult-oriented clinic

#### Bivariable and Multivariable logistic regression analysis

In the bivariable logistic regression analysis, statistical significance was observed among variables such as age at transition, residence, gender, opportunistic infection, duration of time on ART, and follow-up model (***refer to*** Table 2).

Predictors showing an association at a p-value ≤ 0.25 in the bivariable logistic regression were included in the multivariable regression to control for confounding effects. Ultimately, age at transition, residence, opportunistic infection, and duration of time on ART were identified as predictors for successful transition from pediatric to adult-oriented HIV care (refer to Table 2).

Among the study participants, 230 (68.25%) successfully transitioned (95% CI = 63.25, 73.25). Among the study participants, 230 (68.25%) successfully transitioned (95% CI = 63.25, 73.25). Of the 107 unsuccessful transitioned adolescents and young adults, 59 (55%) had both a high viral load and missed at least one scheduled care visit, while the remaining 28 (26%) and 20 (19%) had a high viral load and missed at least one care visit respectively. The odds of a successful transition from pediatric to adult HIV clinic were 4.25 times higher for those transitioning at age 18 or older compared to those transitioning at age less than 18 (AOR = 4.25; 95% CI = 2.29, 7.87; *P* < 0.000). Furthermore, the odds of a successful transition were 1.78 times higher for individuals residing in urban areas compared to those in rural areas (AOR = 1.78; 95% CI = 1.04, 3.02; *P* < 0.033). Individuals with opportunistic infections at the study enrollment had 66% lower odds of a successful transition compared to those without such infections (AOR = 0.34; 95% CI = 0.15, 0.75; *P* < 0.008). Additionally, individuals on ART for more than two years had 2.4 times higher odds of successful transition compared to those on ART for less than one year (AOR = 2.4; 95% CI = 1.17, 4.94; *P* < 0.017).

**Table 2:** Bivariable and multivariable logistic regression analysis for predictors of a successful transition among adolescents and young adults living with HIV who transition from pediatric to adult-oriented HIV care in selected health facilities of southern Ethiopia (*n* = 337)

Variables	Successful transition		Crude odd ratio (COR) with 95% CI	*P*-value	Adjusted odd ratio (AOR) with 95% CI	*P*-value
	Yes	No				
Age at transition(years) < 18 ≥ 18	131 99	89 18	ref 3.73 (2.11,6.60)	0.001*	ref 4.25(2.29,7.87)	< 0.001
Gender Male Female	104 126	58 49	0.69(0.43,1.10) ref	0.125*	0.67(0.40,1.12) ref	0.132
Residence Urban Rural	170 60	60 47	2.21 (1.37,3.59) ref	0.001*	1.78(1.04,3.02) ref	0.033
Alcohol Intake during the study enrollment Yes No	13 217	6 101	1.00 (0.37, 2.72) ref	0.987		
Smoking during the study enrollment Yes No	13 217	5 102	1.22 (0.42, 3.52) ref	0.710		
Opportunistic infections during the study enrollment Yes No	16 214	18 89	0.36 (0.18,0.75) ref	0.007*	0.34(0.15,0.75) ref	0.008
Developmental disorder Yes No	4 226	2 105	0.92 (0.16, 5.15) ref	0.933		
ART regimen First line Second line	207 23	97 10	0.92 (0.42, 2.02) ref	0.851		
Duration on ART < 1 year 1–2 year > 2 year	40 61 129	22 41 44	ref 0.81 (0.42,1.57) 1.61 (0.86,3.00)	0.548 0.133*	ref 0.94(0.45,1.94) 2.40(1.17,4.94)	0.880 0.017
Follow-up model Every 1 month Every 3 month Every 6 month	91 61 78	33 28 46	ref 0.79(0.43,1.43) 0.61(0.35,1.05)	0.441* 0.077*	ref 0.70(0.36,1.36) 0.57(0.31,1.03)	0.297 0.067

ref = reference

* candidate for multivariable logistic regression (P-value < 0.25)

## Discussion

The study aimed to identify predictors of a successful transition from pediatric to adult antiretroviral therapy (ART)-oriented HIV care among adolescents and young adults who underwent transition between January 2017 and August 2022 in health facilities in southern Ethiopia. Approximately 68% (95% CI = 63.25, 73.25) of adolescents and young adults successfully transition from pediatric to adult ART clinics. Age at transition, residence, duration of time on ART, and opportunistic infections were identified as predictors for a successful transition from pediatric to adult ART clinics.

The Ethiopia National Strategic Plan for HIV aims to achieve national control of the HIV epidemic by 2025, aligning with the 95-95-95 targets set by the Joint United Nations Program on HIV/AIDS (UNAIDS) [10]. The primary objective is to reduce new HIV infections and AIDS-related mortality to less than 1 per 10,000 population by attaining 95% viral suppression among People Living with HIV (PLHIV) receiving treatment [35].

However, our study revealed that only 68.25% of HIV patients undergoing treatment successfully transitioned (maintained in care and achieved viral suppression). This suggests a considerable proportion of HIV patients experienced high viral loads, potentially leading to opportunistic infections, AIDS-related morbidity, and mortality. This finding contrasts with studies conducted in Bangkok, Italy, South Africa, and Kenya [36–39], where the success of the transition was higher. This discrepancy may be due to variations in governance, leadership, health expenditure, healthcare workforce, medical product supplies, health service delivery, and health system information, all of which can directly and indirectly influence the transition process.

In Ethiopia, the absence of a structured transition process and plan has hindered patients’ successful transition to adult HIV care. Implementation of the transition process varies across health facilities, lacking a standardized guideline to facilitate the transition [14]. Nevertheless, our results surpass findings from studies conducted in the USA [38, 40]. Differences in the study period, design, population characteristics, and sample size may contribute to this variation. The U.S. studies were conducted on smaller sample sizes, with more than half of the cases behaviorally acquired in the first study and focusing on those transitioning before 20 years in the second study. These differences may affect the rate of successful transition, suggesting potential variations in the quality of the transition process during these specific periods.

In this study, the age of transition emerged as a significant predictor for a successful transition to an adult ART clinic. Patients transitioning at the age of 18 or older were more likely to have a successful transition compared to those transitioning at an age less than 18. This finding aligns with studies conducted in Thailand, Kenya, and South Africa [36, 38, 39]. The observed trend may be attributed to individuals transitioning at or after the age of 18 demonstrating mature concrete thinking, often reflected in a sense of responsibility, vulnerability, and adherence to treatment. Additionally, this age range signifies a crucial stage in a person’s life when they legally assume responsibility for their healthcare decisions. Negotiating the complexities of managing their HIV condition independently, they actively seek appropriate treatment and support tailored to their specific needs. These characteristics collectively may contribute positively to the ability to adhere to complex medical regimens, resulting in a successful transition.

The residential context of adolescents and young adults transitioning from pediatric to adult HIV clinics is a crucial aspect to explore for enhancing healthcare outcomes. In the current study, an examination of urban and rural distinctions revealed that residence significantly influenced the success of transitioning to an adult ART clinic. Notably, adolescents and young adults residing in urban areas demonstrated a higher likelihood of a successful transition compared to their rural counterparts, aligning with findings from a study conducted in North America and Europe [41].

This disparity could be attributed to the easier access urban residents have to health facilities and better quality healthcare services. Additionally, their proximity to media and information related to HIV may contribute to a smoother transition to adult care [42]. In contrast, those from rural areas face distinct challenges accessing HIV clinics, including limited transportation options and a scarcity of healthcare providers specializing in HIV care. They are also more susceptible to stigma, discrimination, and social isolation, factors that increase the likelihood of becoming lost to follow-up and discontinuing HIV care [42].

In this study, the duration of time on ART emerged as another significant predictor for the successful transition of HIV care to an adult ART clinic. Patients who had been on ART for more than two years were more likely to undergo a successful transition compared to those with less than one year on ART, aligning with findings from studies conducted in Kenya and South Africa [39, 43, 44]. This trend could be attributed to the gradual improvement in CD4 count over time after the initiation of ART, subsequently leading to viral suppression and a successful transition. In addition, the long duration of ART provides individuals with more time to mentally adapt to their diagnosis. During this time, they may also pursue mental health resources, such as counseling or support groups, which can facilitate acceptance and offer coping strategies for managing the diagnosis.

Finally, the presence of opportunistic infections has been identified as a hindering predictor for the successful transition to adult-focused HIV care. Patients with OIs demonstrate lower odds of successful transition compared to those without them. This finding is aligned with research findings from Uganda and Ethiopia [45, 46]. Opportunistic infections can worsen HIV replication, resulting in a weakened immune system and higher viral load. Increased HIV replication due to opportunistic infections adds strain to the immune system, making it more difficult for patients to achieve health stability and adhere to necessary treatment regimens for transitioning to adult care. This double effect not only diminishes the body’s ability to defend itself but also complicates the shift to adult-focused HIV care. Furthermore, these infections can result in missed follow-up appointments and significant health issues spanning from mild to severe impacting organ systems and overall well-being. This complexity makes it harder for individuals to effectively manage their HIV during this phase.

Additionally managing both HIV and opportunistic infections demands adherence to medication schedules and medical visits. The shift from pediatric to adult care facilities may disrupt established routines and support systems potentially leading to gaps, in treatment compliance and heightened risks of disease advancement.

## Limitations of the study

Despite the extensive scale and prolonged follow-up duration of this study, along with efforts to encompass all transitioned patients in selected health facilities, certain limitations should be acknowledged. Firstly, the data derived from records exhibited some missing values, and critical variables such as knowledge and attitude about transition, food insecurity, stable housing, education attained, in school or out of school, living alone or in a family, household income, service satisfaction, risky sexual behavior, and psychosocial support were not considered in the study. Secondly, the absence of a standardized definition for successful transition complicates comparisons with other studies. Thirdly, the study design limits the ability to confirm associations between transition practices and the examined variables, while a randomized trial would be ideal for establishing causality. Finally, the sample size of this study is relatively small, which may impact the generalizability of the findings.

## Conclusion

Less than 70% of HIV patients receiving care successfully transitioned in South Ethiopia, falling below the targets set by UNAIDS in 2014 [9] and the Ethiopian national strategic plan [23]. Positive predictors for successful transition included age at transition equal to or greater than 18 years, urban residence, and a duration of time on ART equal to or greater than 2 years. Conversely, having opportunistic infections was identified as a negative predictor for the successful transition of HIV care from pediatric to adult HIV clinic. To ensure successful transitions of adolescents and young adults living with HIV from pediatric to adult care, it’s crucial to implement a standardized transition plan tailored to address transition age and urban and rural disparities. This plan should prioritize early initiation of ART, integrate mental health support, establish robust monitoring systems, and emphasize infection prevention. Improving data quality will further enhance evidence-based practices, ultimately optimizing the transition process for this vulnerable population.

## Data Availability

The datasets used during the current study are available from the corresponding author upon reasonable request.
